# Balance Performance in People With Peripheral Visual Field Loss: A Systematic Review and Meta-Analysis

**DOI:** 10.1167/tvst.15.7.10

**Published:** 2026-07-08

**Authors:** Peng Yi Hong, Jia Shu Wen, Chan Marco Lok Hin, Stanley Winser, Mark Oremus, Benjamin Thompson, Cheong Allen Ming Yan

**Affiliations:** 1 School of Optometry, The Hong Kong Polytechnic University, HKSAR; 2 Department of Rehabilitation Sciences, The Hong Kong Polytechnic University, HKSAR; 3 School of Public Health Science, University of Waterloo, Canada; 4 School of Optometry and Vision Science, University of Waterloo, Canada; 5 Centre for Eye and Vision Research Limited, Hong Kong Science Park, HKSAR; 6 Research Centre for SHARP Vision, The Hong Kong Polytechnic University, HKSAR

**Keywords:** balance control, postural control, fall

## Abstract

**Background:**

Although peripheral visual field loss (PVFL) affects balance, previous research has produced mixed results and has not clearly distinguished the independent roles of different systems in balance impairment. This systematic review with meta-analysis is the first to comprehensively evaluate the proportion and contribution of different systems to balance in adults with PVFL, providing new insights for rehabilitation strategies.

**Methods:**

A systematic search was performed in five databases (PubMed, AMED, Scopus, CINAHL, and Web of Science) from 2003 to 2023, with a manual search covering 2024 to 2025. The review focused on observational studies in adults (≥18 years) with PVFL because of glaucoma or retinitis pigmentosa. Data were synthesized by summarizing the balance function into two aspects: sensory strategy and motor strategy. The sensory strategy was defined as the motor response to changes in altering visual, vestibular, or somatosensory inputs to maintain balance, whereas the motor strategy was defined as the response to other conditions besides visual, vestibular, or somatosensory conditions, such as response to external perturbation, muscle coordination, and reaction time. The meta-analysis was conducted where appropriate.

**Results:**

Seventeen studies comprising a total of 2456 participants were analyzed. The meta-analysis revealed that individuals with PVFL exhibited poorer balance than age-matched controls. Specifically, PVFL was associated with a reduced contribution from the visual system, an increased contribution from the somatosensory system, and poorer motor strategies in maintaining balance. Greater peripheral visual field loss correlated with poorer sensory strategy for balance, whereas the relationship between motor strategy and peripheral visual field loss remained inconclusive.

**Conclusions:**

Adults with PVFL demonstrate significant balance impairments compared to healthy peers, relying more on somatosensory input to compensate for visual deficits. These findings underscore the need for comprehensive balance assessments and targeted rehabilitation strategies. Further research should focus on identifying the most predictive balance parameters and developing targeted, evidence-based rehabilitation strategies for this population.

**Translational Relevance:**

By identifying the specific sensory-motor mechanisms of balance impairment in patients with PVFL, this study bridges the gap between clinical vision assessment and rehabilitation. The finding that these patients rely heavily on somatosensory compensation provides a strong clinical rationale to transition from generic balance exercises to personalized, targeted rehabilitation strategies (such as proprioceptive and motor-strategy training), ultimately reducing fall risks and improving mobility in patients with PVFL.

## Introduction

Balance control is a complex function that relies on the integration of sensory inputs, neuromuscular coordination, and musculoskeletal integrity.^1^ Sensory contributions to balance arise from the vestibular, somatosensory, and visual systems. The vestibular system provides essential information about head position and movement in space,^2^ whereas the somatosensory system conveys internal body status. The visual system, which is sensitive to the central nervous system processing, assists in postural adjustments by integrating visual perception and spatial awareness.^3^ Neuromuscular interactions between the central and peripheral nervous systems and the musculature govern posture and movement.^4^ The musculoskeletal system contributes through muscle strength, joint stability, postural alignment, movement control, flexibility, and endurance.^5^

Impairment in any of these systems can compromise balance control. For example, stroke survivors may experience trunk muscle weakness, leading to impaired standing and sitting balance.^6^ Similarly, individuals with osteoarthritis may lose neuromuscular function, increasing the risk of falling and fall rates.^7^ Visual impairments can disrupt sensory interactions, altering the perception of body position and thereby affecting balance.^8^ Thus visual input is essential for maintaining postural stability.

The visual system comprises the central and peripheral visual fields. The central field is responsible for high-resolution vision and feature identification, such as color and shape, whereas the peripheral field is crucial for detecting motion and spatial dynamics, including the movement and position of surrounding objects.^9^ Some hypotheses suggest that the peripheral visual field plays a more significant role in regulating anterior-posterior adjustments than the central field,^10^^,^^11^ while others propose that it has a greater influence on overall postural stability.^12^ Collectively, these perspectives underscore the importance of the peripheral visual system in balance control.

Evidence indicates that individuals with peripheral visual field loss (PVFL) are more susceptible to falls and impaired balance, as evidenced by increased center of pressure sway area and path—compared to age-matched healthy counterparts.^13^^–^^16^ However, findings across studies are inconsistent, likely because of variations in assessment methods, including different approaches to detecting the sensory system inputs or muscle strength. This methodological heterogeneity has led to divergent conclusions regarding the extent and nature of balance impairments in PVFL, highlighting the need for standardized assessment tools.^13^^,^^14^^,^^17^^–^^19^

To our knowledge, no systematic reviews or meta-analyses to date have ever comprehensively examined the impact of PVFL on balance function, nor have they summarized the contributions of sensory and motor aspects to balance control in PVFL. Therefore we conducted a comprehensive review and meta-analysis to elucidate the effect of PVFL on balance through these two aspects and to explore the relationship between balance control and peripheral visual field deficits.

## Methods

This systematic review followed the Preferred Reporting Items for Systematic Reviews and Meta-analyses (PRISMA) 2020 guidelines (Supplementary Material S1)^20^ and was registered with the International Prospective Register of Systematic Reviews in November 2023 (PROSPERO; Ref. No: CRD42023483578). The research questions were formulated using the PECOS (Population, Exposure, Comparison, Outcome, and Study type) format, focusing on observational study designs. Compared to healthy controls, this review focused on adults (age ≥ 18 years) with PVFL caused by glaucoma or retinitis pigmentosa. No interventions were included in this review because the baseline data from intervention studies lacked any analytical comparisons with healthy controls and did not investigate the relationship between PVFL and balance. Outcomes assessed by various balance measures, including sensory systems’ inputs detection assessments (e.g., Clinical Test of Sensory Interaction on Balance [CTSIB], Modified Clinical Test of Sensory Interaction on Balance [mCTSIB], Sensory Organization Test [SOT]) and motor systems’ inputs detection assessments (e.g., Timed Up and Go Test [TUG], Motor Control Test (MCT), Standing balance test). The review protocol was conceptualized and developed by authors AYHP, SW, and AMYC.

### Search Strategy

We searched five databases - PubMed, AMED, Scopus, CINAHL, and Web of Science- for relevant studies published between January 2003 and December 2023. This 20-year timeframe was chosen to capture recent and relevant research, reflecting current trends and advancements. Keywords were selected from MeSH terms and adapted to maximize coverage. The search themes were divided into two groups: (a) Peripheral visual field loss (e.g., peripheral visual field loss, vision loss, peripheral visual field damage, visual field defect, glaucoma, retinitis pigmentosa and RP; and (b) Balance performance (e.g., balance, postural sway, postural control, postural stability, stabilization, equilibrium, mobility, fall). The Boolean operator “AND” was used to combine the two groups, whereas “OR” was used within each group. A citation management software (ENDNOTE 21, Clarivate Analytics, Philadelphia, PA, USA) was used to organize the search results and remove duplicates. Manual searches were also conducted. Two authors (AYHP and SWJ) independently conducted the literature search. Details of the PubMed search strategy are provided in Supplementary Material S2. A secondary search was conducted in January 2025 to identify new literature published between January 2024 and December 2024.

### Study Eligibility Criteria

Studies were included if they (a) reported quantitative results from balance tests in individuals aged 18 years or older with PVFL because of glaucoma or retinitis pigmentosa; (b) were published in English or Chinese; (c) presented the results of comparisons or associations between visual field and balance function from observational studies (e.g., cross-sectional, cohort, case-control designs); (d) were available in full text; (e) were published between 2003 to 2023. Exclusion criteria were: (a) randomized controlled trials, published protocols, conference abstracts, preprints, and review articles; (b) studies involving healthy participants with simulated visual field loss; and (c) studies including subjects with congenital blindness or other conditions affecting the visual field, such as cataracts, age-related macular disease, stroke, or Parkinson's disease.

### Article Screening

Two authors (AYHP and SWJ) independently screened the titles and abstracts. Full texts of potentially eligible articles were independently reviewed by two authors (AYHP and LHC). Discrepancies were resolved by discussion, or if necessary, were resolved by consulting a third author (AMYC).

### Data Extraction

The primary outcome was balance measurement, including tests such as CTSIB, mCTSIB, SOT, TUG, MCT, and the standing balance test (Refer to Supplementary Material S3). Two authors (AYHP and LHC) independently extracted data using a custom Microsoft Excel tool, including author details, year, participant characteristics, visual assessments, balance assessment methods, and conclusions. Disagreements were resolved by a third author (AMYC).

### Quality Appraisal

Two authors (SWJ and LHC) independently appraised the quality of the included studies using the Joanna Briggs Institute critical appraisal tool.^21^ This tool was appropriate for cross-sectional, cohort, and case-control studies. The interrater reliability for the risk of bias assessments was 90.9%, with a kappa statistic of 0.75. Disagreements were resolved by consulting a third author (AMYC).

### Data Synthesis and Statistical Analysis

Balance control involves complex interactions across different systems. We summarized these complex interactions into two aspects: the sensory strategy and the motor strategy.^22^^,^^23^ The motor response to changes in the sensory systems’ inputs, involving the visual, vestibular, and somatosensory systems, is one crucial aspect for maintaining balance.^24^ In our review, we defined it as “sensory strategy.” Clinical assessments to evaluate the sensory strategy for PVFL often utilize force plates with unstable surface conditions (e.g., foam surfaces) and variable visual conditions (e.g., eyes closed or virtual reality) to assess. We categorized those assessments into three scenarios: (1) somatosensory perturbation, where the situation that subjects stand on foam or unstable surfaces to reduce somatosensory system's input; (2) visual perturbation, where the situation that subjects close their eyes on a firm surface to assess visual system's input; and (3) simultaneous perturbation, where the situation that subjects stand on foam or unstable surfaces with eyes closed to evaluate vestibular system's input. The motor responses to other conditions besides visual, vestibular, or somatosensory conditions, such as neuromuscular responses to the external environment, muscle coordination, reaction times, and movement strategies, were grouped under another aspect, “motor strategy.”^25^ It is assessed by tests such as the MCT, Balance Self-efficacy Scale, Standing Balance Test, and TUG test.

Both sensory strategy and motor strategy were summarized and analyzed in our systematic review to provide a comprehensive view of balance control. A narrative synthesis was first conducted following the guidelines from the Centre for Reviews and Dissemination.^26^ Balance outcome parameters including root mean square sway, sway velocity, SOT Score, and standard deviations of the torque moments from studies involving individuals with PVFL and age-matched healthy controls (HC),^13^^–^^15^^,^^19^^,^^27^^–^^30^ as well as sensory system contribution ratios from studies selected,^15^^,^^19^^,^^28^^,^^29^ were pooled for meta-analysis. Statistical heterogeneity was assessed using the *I^2^* index,^31^ and results were pooled using fixed-effect or random-effect models according to the level of heterogeneity. Hedges’ g was used to calculate bias-corrected effect sizes, quantifying the standardized mean differences. Egger's regression test was used to examine publication bias (except when fewer than 10 studies were included). The Comprehensive Meta-analysis software (CMA version 3.0, Biostat Inc., Englewood, NJ, USA) was used for the meta-analysis.

## Results

### Study Selection

**Figure 1.**
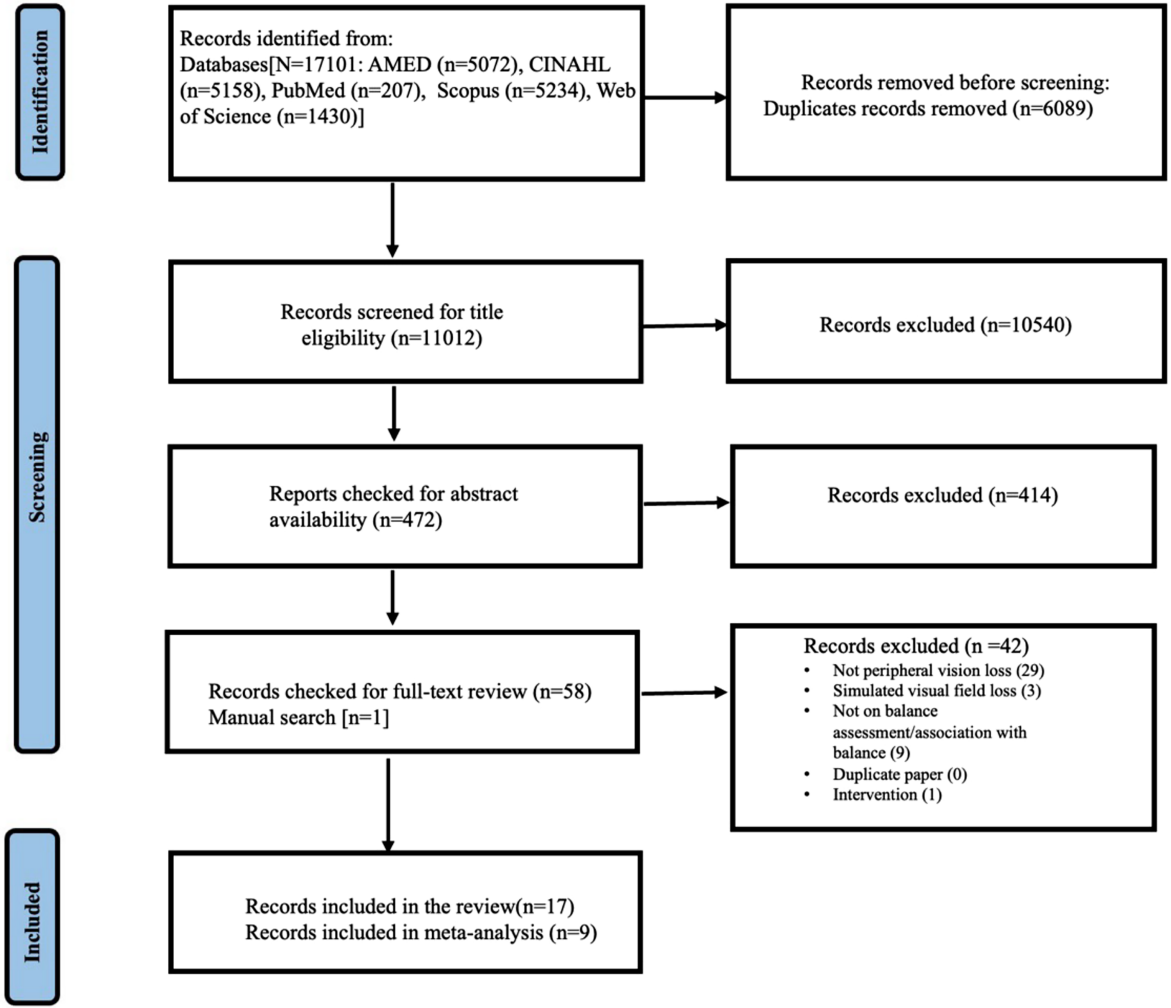
Study flowchart.

A total of 17,101 records were identified from five databases. After removing 6089 duplicates, 11,012 unique records remained for screening. Of these, 10,540 were excluded based on title, leaving 472 records for abstract review. After screening abstracts, 58 studies were selected for full-text review, of which 42 were excluded for not meeting eligibility criteria. One additional study, identified through manual search and published after December 2023 or previously omitted, was included. In total, 17 studies comprising 2456 participants were included in this systematic review (Fig. 1).

### Characteristics of Included Studies and Participants

The 17 included studies involved 2456 participants, with mean ages ranging from 48^29^ to 79^32^ years. Most samples consisted of older adults. The sex distribution was uneven, with more women (*n* = 1240) than men (*n* = 1082), excluding two studies where sex was not specified.^15^^,^^29^ All participants had PVFL attributed to glaucoma or RP.

Nine studies investigated the relationship between PVFL and balance function,^15^^,^^29^^,^^30^^,^^33^^–^^38^ whereas 10 studies compared balance function between individuals with PVFL and those with normal vision.^13^^–^^16^^,^^19^^,^^27^^–^^30^^,^^32^ Detailed study characteristics are presented in Table 1.

**Table 1.** Characteristics of the Included Studies

		Visual Assessments		Balance Assessments			
Study Reference	Participant Characteristics Number, Sex Distribution (M/F), Age	Visual Acuity (logMAR) & Contrast Sensitivity	Visual Field	Method/Tool	Set Up/Condition	Parameters	Conclusions
Black 2008	PVFL: • 54 Glau; • 33/21; • 74.4 ± 5.8	BE BCVA (median):0.05 (−0.26-0.52)BE CS (median):1.60 (0.65 – 1.70)	HFA 24–2 SITA-Standard test (median): • VF60: −2.50 dB (+1.60 to −28.00) • Binocular VF120, no. of median points missed: 28 (6-96) • VF120 Hemifield difference score (%): 4 ± 16	Sway meter	Total sway area in 30s under the following 4 conditions.:(i) eyes open, firm surface;(ii) eyes closed, firm surface;(iii) eyes open, foam surface;(iv) eyes closed, foam surface	• Sway area• Visual stability ratio	• Greater binocular visual field loss showed increased postural sway, both on firm and foam surfaces. • Greater inferior hemifield visual field loss showed increased postural sway on the foam surface. • Increased glaucomatous visual field loss was accompanied by a steady linear decrease in the visual contribution to postural control. • Visual loss measures were significant predictors of postural sway, explaining almost 20% of its variance on the foam surface.
Black 2011	PVFL: • 74 Glau; • 39/35; • 74.2 ± 5.9	BE BCVA:0.06 ± 0.13BE CS:1.54 ± 0.17	HFA 24-2 SITA-Standard test: • IVF-60 VF: −4.10 ± 6.28 • IVF-120 VF, points missed: 32 ± 21	—	TUG test	TUG time	• Poorer visual field was associated with slower TUG performance. • Inferior visual field and contrast sensitivity were the strongest predictors of balance outcomes, whereas the superior visual field factor was not related to TUG performance.
Cadoni 2022^*^	PVFL: • 8 PVFL; • 6/2; • 59 ± 12.7 Control: • 10 HC; • 5/5; • 57 ± 11.2	BE Habitual VA: • PVFL: 0.5 ±0.3; • HC: — WE Habitual VA: • PVFL: 0.6 ±0.6; • HC: — CS: —	HFA II: • Mean peripheral binocular VF (%): 12.2 ± 7.7	• SOT: Equitest, Neurocom Int. Inc., OR, USA • MCT: Equitest, Neurocom Int. Inc., OR, USA	• SOT • MCT	• SOT: (i) the equilibrium score (ES);(ii) Composite Equilibrium Score (CES);(iii) Sensory Analysis (SA) • MCT: (i) Weight Symmetry (WS);(ii) Latency;(iii) Composite Latency Score (CLS);	• The PVFL group showed poorer performance compared to the HC group in the SOT. • No significant difference was found at somatosensory contribution in SOT between groups, visual afferences were notably higher in the HC group. • Apart from medium forward and backward translations on the left leg in MCT, no statistical differences were found between the HC and PVFL groups.
Connell 2021	PVFL: • 11 Glau; • 6/5; • 65 ± 9	—	HFA 24-2 SITA-standard test:(i) BE: 2.4 ± 3.7;(ii) WE: − 7.7 ± 7.1	• SOT: Equitest, Neurocom Int. Inc., OR, USA	Adapted SOT with 6 postural conditions, each last 3 min:(i) floor fixed/sway-referenced;(ii) eyes open/closed;(iii) visual scene fixed/sway referenced.	• AP COP RMS: filtered COP displacement • AP COP NPL: time-normalized path length of the COP data	• Visual field deficit severity in the BE was associated with increased standing sway speed. This finding was confirmed in eyes open and closed conditions. • The SOT result was not influenced by the extent of the visual field deficit in the WE, nor was it associated with structural damage in either eye.
de Luna 2017	PVFL: • 236 Glau; • 120/116; • 71	VA:—CS:—	HFA 24-2 SITA standard test:(i) Central VF: • Binocular: 27.1 ± 4.3; • BE:-4.5 ± 6.5; • WE: −8.5 ± 8.2 (ii) Peripheral VF: • BE Mean points missed:13.2 ± 12.5	• the Opal Kinematic system: APDM, Inc., Portland, OR	• mCTSIB • Standing Balance Test: (i) feet together; (ii) feet in a semi-tandem position; (iii) feet in a tandem position	• RMS • Jerk • Visual dependence-Ratio between eyes closed ellipse sway to eyes open ellipse sway	• Lower IVF sensitivity was significantly associated with greater RMS sway during eyes-open foam-surface testing, but not during other ICTSIB conditions. • Lower IVF sensitivity also was significantly associated with greater RMS sway during feet together standing balance testing, but not during other standing balance conditions. • Visual dependence of balance was lower in patients with worse IVF sensitivity.
Diniz-Filho 2015^*^	PVFL: • 42 Glau; • 25/17; • 69.1 ± 11 Control: • 38 HC; • 16/22; • 65.4 ± 11.7	Binocular Habitual VA: • PVFL: −0.03 ± 0.11; • HC: −0.08 ± 0.10CS:—	HFA SITA Standard 24-2 test:(i) WE: • PVFL: −8.0 ± 5.7; • HC: −1.0 ± 1.6. (ii) BE: • PVFL: −3.0 ± 3.5; • HC: 0.0 ± 1.4. (iii) Binocular VF: • PVFL:27.8 ± 3.0; • HC: 31.0 ± 1.8	• Oculus Rift: Oculus VR, LLC, Irvine, CA; • force platform: AMTI Optima Human Performance System, Advanced Mechanical Technology, Inc., Watertown, MA	Postural stability test under 4 conditions:(i) No Oculus Rift;(ii) Oculus Rift in a dark field, without any visual stimulation;(iii) Oculus Rift with rotational stimulus;(iv) Oculus Rift with translational stimulus.	the SDTM of overall, AP, ML	• PVFL had larger overall SDTM than HC during both translational as well as rotational stimuli. • In multivariable model, the postural metrics obtained during dynamic visual stimuli performed better in explaining history of falls compared to those obtained in static and dark field condition. • Higher ML SDTM was significantly associated with more history of falls.
Finger 2016	PVFL: • 40 RP; • 21/19; • 53 ± 16;	Binocular Habitual VA:2.3 ± 1.0CS:—	Manual kinetic Goldmann perimetry:VF Remaining 11.8 ± 20.4	—	Timed up and GO-Low Vision (TUG-LV):(i) extends the course from 3 to 6 meters;(ii) includes bright lights at each end	TUG-LV time	• TUG-LV task time was associated with visual acuity. • The VF was not associated with TUG performance.
Friedman 2007	PVFL: • 1250 Glau; • 513/737; • 79 ± 4.4	Binocular Habitual VA: • Bilateral PVFL: 0.18 ± 0.35; • Unilateral PVFL: 0.11 ± 0.26; • No or Possible PVFL: 0.06 ± 0.22CS: • Bilateral PVFL: 28.67 ± 7.53; • Unilateral PVFL: 31.43 ± 5.00; • No or Possible PVFL: 33.23 ± 4.37	Central VF-the HFA 24-2 SITA fast: • Bilateral PVFL Group: −11.69 ± 8.38; • Unilateral PVFL Group: −6.90 ± 6.04; • No or Possible PVFL Group: −3.46 ± 5.11. Peripheral VF-suprathreshold pattern with a 24-decibel stimulus: • Bilateral PVFL Group: 48.21 ± 23.68; • Unilateral PVFL Group: 35.14 ± 17.48; • No or Possible PVFL Group: 28.05 ± 14.15	—	Three 30-second timed stands under 3 conditions: (i) Semi-tandem: heel of one foot placed at the heel of the first metatarsal phalangeal joint of the other foot;(ii) side-by-side: feet next to each other;(iii) tandem stance (heel of one foot placed at the tip of the first toe of the other foot).	The Failure times of stands	• Bilateral glaucoma enhanced the failure times of stands under three conditions.
Gomes 2018^*^	PVFL: • 33 Glau; • 11/22; • 68.4 ± 8; Control: • 34 HC; • 7/27; • 69.3 ± 7.9	Binocular Habitual VA: • PVFL: 0.07 ± 0.18; • HC: 0.01 ± 0.02CS:—	The Octopus 1-2-3 test:(i) WE: • PVFL: 6.3 ± 3.7; • HC: —; (ii) BE: • PVFL: 4.8 ± 1.8; • HC: —.	—	• TUG test; • Postural sway test: A foam rubber mat with a belt connected to a fixed sway meter that has a pen attached to its end. The pen registered the sway over a millimeter paper placed on top of the adjusted table.	• TUG: TUG time • Postural sway test: (i) Sway area (ii) Total sway path (iii) AP sway displacement (iv) ML sway displacement	• PVFL group presented significantly worse TUG performance compared to the HC group. • PVFL group presented significantly higher risk of falling compared to the HC group. • Individuals in the early and moderate stages of primary open glaucoma presented mobility and sensory deficits that increase the risk of falling.
Kotecha 2012*	PVFL: • 24 Glau; • no sex distribution; • 65.9 ± 5.5 Control: • 24 HC; • no sex distribution; • 68.3 ± 5.2	BE Habitual VA (median): • PVFL:0.02 (0.00 to 0.10); • HC:0.00 (−0.06 to 0.04); WE Habitual VA (median): • PVFL:0.18 (0.03 to 0.29); • HC:0.04 (−0.02 to 0.16); Binocular Habitual VA (median): • PVFL:0.00 (−0.08 to 0.08); • HC:0.00 (−0.1 to 0.04). BE CS (median): • PVFL:1.5 (1.36 to 1.5) • HC:1.65 (1.6 to 1.65) WE CS (median): • PVFL:1.45 (1.36 to 1.5) • HC:1.6 (1.50 to 1.65) Binocular CS (median): • PVFL:1.60 (1.39 to 1.65) • HC:1.65 (1.65 to 1.73)	HFA SITA Standard 24-2 test:(i) BE (median): • PVFL: −7.30 (−14.88 to −3.37); • HC: 0.42 (−0.41 to 1.12); (ii) WE (median): • PVFL: −15.19 (−19.91 to −9.42); • HC: −0.35 (−0.83 to 0.58)	Force-balance platform:Bertec Corporation, Columbus, OH;	30-second stand period under 4 conditions with repeated 3 times:(i) eyes open on the firm surface;(ii) eyes close on the firm surface;(iii) eyes open on the foam rubber surface;(iv) eyes close on the foam rubber surface	• AP RMS sway • ML RMS sway • AP sway velocity • ML sway velocity • RQ : sway velocity ECFo/sway velocity EOFo	• PVFL had a lower visual contribution to sway, and higher relative somatosensory contribution to sway. • Binocular MD was a significant predictor of balance.
Kotecha 2013^*^	PVFL: • 12 Glau; • 6/6; • 69.2 ± 4.3; Control: • 12 HC; • 8/4; • 66.2 ± 6.4	Binocular Habitual VA: • PVFL: 0.06 ± 0.13; • HC: −0.02 ± 0.11 Binocular CS: • PVFL: 1.46 ± 0.18 • HC: 1.7 ± 0.08	HFA SITA standard 24-2 test:BE: • HC:0.44 ± 1.10; • PVFL: −10.76 ±0.50.	Force-balance platform:Bertec Corporation, Columbus, OH;	Stand on the center of the platform with 4 conditions:(i) firm surface/foam rubber surface;(ii) quiet stance/whilst performing a mental arithmetic task	• RMS AP COP • RMS ML COP	• The difference between HC and PVFL approached significance when undertaking the mental arithmetic task on the foam surface.
Kotecha 2016^*^	PVFL: • 11 Glau; • 4/7; • 51.6 ± 5.3 Control: • 11 HC; • 5/6; • 49.7 ± 5.3	Binocular Habitual VA: • PVFL: 1.8 (range 1.0 to no-light perception with severe constriction of visual fields)CS:—	—	Bertec force balance platform:Bertec Corporation, Columbus, OH, USA.	12 randomly experimental conditions include:(i) eye conditions:eyes open/eyes close(ii) surface conditions: firm platform/ foam platform (iii) touch conditions: no touch/light touch/unrestricted touch	• RMS COP; • RQ: Balance Eyes Closed Foam/Balance Eyes Open Foam; • SR: Balance Eyes Open Foam/Balance Eyes Open Firm	• PVFL had a significantly increased SR compared to HC. They had a significantly lower RQ compared to HC. • There was a significant effect of touch, vision, and surface on balance control. • The effects of touch and vision were greater in HC when their vision was removed, and greater in patients when their somatosensory system was disrupted.
Mihailovic 2020	PVFL: • 239 Glau; • 124/115; • 70.5 ± 7.6	BE Habitual VA (median):0.06 (−0.02, 0.16)Binocular CS (median):1.72 (1.64, 1.76)	Humphrey Field Analyzer II:(i) Binocular VF (median): • All subjects: 28.0 (26.0, 29.7); • Manifest PVFL: 27.7 (25.8, 29.6).(ii) BE (median):−2.6 (–5.4, −0.7);(iii) WE (median):−5.7 (−12.9, −2.8)	The Opal Kinematic system:APDM, Inc., Portland, OR, USA	ICTSIB:stand still on the foam surface with eyes open for 30 seconds.	• RMS sway; • total sway • ellipse sway • Jerk	• Worse balance was associated with a higher rate of falls per year and step. • No balance measures mediated the relationship between visual field damage and fall rates. IVF remained an independent predictor of falls per step in multivariable models including individual balance parameters.
Popescu 2011	PVFL: • 82 Glau; • 46/36; • 76.5 ± 7.4 Control: • 73 HC; • 28/45; • 72.8 ± 4.6	Binocular Habitual VA: • PVFL: 0.33 ± 0.32; • HC: 0.04 ± 0.06;WE CS: • PVFL: 0.85 ± 0.56; • HC: 1.72 ± 0.17.	Humphrey Full threshold N-30 test: BE: • PVFL: −9.6 ± 6.6; • HC: 0.5 ± 2.0.	—	(i) TUG test;(ii) one-legged balance test: stand on the leg of choice with eyes open for up to 30 seconds	• TUG times • The availability of one single leg test	• PVFL group had worse TUG scores and were more likely to have poor balance than the HC group.
Shabana 2005^*^	PVFL: • 21 Glau; • no sex distribution; • 52 Control: • 35 HC; • no sex distribution; • 48	—	HFA 24-2 SITA-standard test: Results: —	Kistler 9281B: with four piezoelectric transducers	Stand still with barefoot, feet separated by 30 angle and heels placed 5cm apart with 4 visual conditions:eyes open/eyes closed/right eye open/left eye open.	• COP velocity (AP, ML) • stabilization ratio: SR(V) = 1 - Log(V(open) + 1)/Log(V(EC) + 1) cm	• For all subjects, sway velocity was lower with vision, highlighting a visual contribution to posture across all stages of glaucoma. This contribution was significantly reduced in POAG patients compared to normal, and further decreased with worsening MD or higher AGIS scores. • The MD of the WE showed the strongest negative correlation with visual contribution to posture, while POAG patients exhibited a greater somatosensory contribution to postural steadiness compared to normal, regardless of monocular or binocular vision.
Tokunaga 2024	PVFL: • 30 Glau; • 15/15; • 64.5(median)	OS BCVA (median):1.2(1.0-1.2);OD BCVA (median):1.2(1.0-1.5)CS:—	HFA SITA standard 24-2 test:(i) OD (median): −2.37 (−5.38 to −1.09) (ii) OS (median): −2.70 (−5.21 to −0.67)	—	• Standing test with 2 conditions: solid/rubber foam surface • ABC scale	• The foam ratio • Visual/somatosensory ratio	• No significant correlation was observed between visual acuity or field deficits and body equilibrium function or fall risk. • Greater peripheral visual field impairment was associated with a tendency for sensory reweighting from visual to somatosensory.
Zwierko 2021^*^	PVFL: • 19 Glau; • 9/10; • 70.6 ± 3 Control: • 19 HC; • 8/11; • 68.8 ± 2.7	BE BCVA (Snellen): • PVFL: 0.8 ± 0.2 • HC: 0.7 ± 0.2	HFA 24-2 SITA-standard test: (i) BE: • PVFL: −7.32 ± 5.35; • HC: 0.81 ± 0.83; (ii) WE: • PVFL: −16.36 ± 7.83; • HC: 0.33 ± 0.94; (iii) Binocular VF: • PVFL: 29.52 ± 19.38; • HC: 0.47 ± 0.09	the Biodex Balance System SD:Biodex Medical Systems Inc., Shirley, NY	• Postural stability test: Stand on the static balance platform with 80s (3 trials of 20s each, with a rest interval of 10s between each) • Fall risk test: Balance on the unstable platform	• AP index • ML index • overall stability index • Fall risk index	• PVFL showed poorer values for most of the mobility and balance control parameters with medium and large effect size. • Mobility scores in PVFL were partly associated with their monocular visual field defect. • Low physical activity was identified as a risk factor for falls and postural instability. Functional declines in dynamic tasks were not related to glaucoma severity.

ABC scale, Activities-specific Balance Confidence scale; AMD, Age-related Macular Disease; AP, Anterior-Posterior; BCVA, best corrected visual acuity; BE, better eye; BES, Balance self-Efficacy Scale; COP, center of pressure; DB, decibel; Glau, glaucoma; HC, healthy control; HFA, Humphrey field analyzer; IVF, integrated visual field; MD, mean deviation; ML, Medio-Lateral; OD, right eye; OS, left eye; RMS, root mean square; RP, retinitis pigmentosa; RQ, Romberg quotient; SDTM, standard deviations of the torque moments; TUG, Time Up and Go test; WE, worse eye.

* The papers picked up for meta-analysis.

### Methodological Quality

The methodological quality of the included studies is summarized in Table 2A, 2B and 2C. Cross-sectional studies scored between 6^37^ and 8 out of 8, the cohort study scored 11 out of 11, and case-control studies scored 10 out of 10. Most studies clearly defined inclusion criteria, study subjects, and setting, and used valid and reliable outcome measures. However, two studies did not adequately identify confounding factors,^14^^,^^37^ and one study lacked a clear description of the exposure.^28^

**Table 2A.** Study Quality Rating: Cross-Sectional

Authors & Year	1. Were The Criteria for Inclusion in the Sample Clearly Defined?	2. Were the Study Subjects and the Setting Described in Detail?	3. Was the Exposure Measured in a Valid and Reliable Way?	4. Were Objective, Standard Criteria Used for Measurement of the Condition?	5. Were Confounding Factors Identified?	6. Were Strategies to Deal With Confounding Factors Stated?	7. Were the Outcomes Measured in a Valid and Reliable Way?	8. Was Appropriate Statistical Analysis Used?	Score (8)
Black 2008	Yes	Yes	Yes	Yes	Yes	Yes	Yes	Yes	8
Black 2011	Yes	Yes	Yes	Yes	Yes	Yes	Yes	Yes	8
Connell 2021	Yes	Yes	Yes	Yes	No	No	Yes	Yes	6
deluna 2017	Yes	Yes	Yes	Yes	Yes	Yes	Yes	Yes	8
Diniz 2015	Yes	Yes	Yes	Yes	Yes	Yes	Yes	Yes	8
Finger 2016	Yes	Yes	Yes	Yes	Yes	Yes	Yes	Yes	8
Friedman 2007	Yes	Yes	Yes	Yes	Yes	Yes	Yes	Yes	8
Gomes 2018	Yes	Yes	Yes	Yes	Yes	No	Yes	Yes	7
Kotecha 2012	Yes	Yes	Yes	Yes	Yes	Yes	Yes	Yes	8
Kotecha 2016	Yes	Yes	Unclear	Yes	Yes	Yes	Yes	Yes	7
Popescu 2011	Yes	Yes	Yes	Yes	Yes	Yes	Yes	Yes	8
Tokunaga 2024	Yes	Yes	Yes	Yes	Yes	Yes	Yes	Yes	8
Zwierko 2021	Yes	Yes	Yes	Yes	Yes	Yes	Yes	Yes	8

**Table 2B.** Study Quality Rating: Cohort

Authors & Year	1. Were the Two Groups Similar and Recruited From the Same Population?	2. Were the Exposures Measured Similarly to Assign People to Both Exposed and Unexposed Groups?	3. Was the Exposure Measured in a Valid and Reliable Way?	4. Were Confounding Factors Identified?	5. Were Strategies to Deal With Confounding Factors Stated?	6. Were the Groups/Participants Free of the Outcome at the Start of the Study (or at the Moment of Exposure?	7. Were the Outcomes Measured in a Valid and Reliable Way?	8. Was the Follow-Up Time Reported and Sufficient to Be Long Enough for Outcomes to Occur?	9. Was Follow-Up Complete, and if Not, Were the Reasons for Loss to Follow-Up Described and Explored?	10. Were Strategies to Address Incomplete Follow-Up Used?	11. Was Appropriate Statistical Analysis Used?	Score (11)
Mihailovic 2020	Yes	Yes	Yes	Yes	Yes	Yes	Yes	Yes	Yes	Yes	Yes	11

**Table 2C.** Study Quality Rating: Case Control

Authors and Year	1. Were the Groups Comparable Other Than the Presence of Disease in Cases or the Absence of Disease in Controls?	2. Were Cases and Controls Matched Appropriately?	3. Were the Same Criteria Used for Identification of Cases and Controls?	4. Was Exposure Measured in a Standard, Valid, and Reliable Way?	5. Was Exposure Measured in the Same Way for Cases and Controls?	6. Were Confounding Factors Identified?	7. Were Strategies to Deal With Confounding Factors Stated?	8. Were Outcomes Assessed in a Standard, Valid and Reliable Way for Cases and Controls?	9. Was the Exposure Period of Interest Long Enough to Be Meaningful?	10. Was Appropriate Statistical Analysis Used?	Score (10)
Cadoni 2022	Yes	Yes	Yes	Yes	Yes	Yes	Yes	Yes	Yes	Yes	10
Kotecha 2013	Yes	Yes	Yes	Yes	Yes	Yes	Yes	Yes	Yes	Yes	10
Shabana 2005	Yes	Yes	Yes	Yes	Yes	Yes	Yes	Yes	Yes	Yes	10

### Data Synthesis and Meta-Analysis

To address our aims, we present results for both sensory strategy and motor strategy aspects, followed by analyses of their associations with visual field loss.

### Balance Control In People With PVFL Versus a Normal Visual Field

#### Sensory Strategy

**Figure 2.**
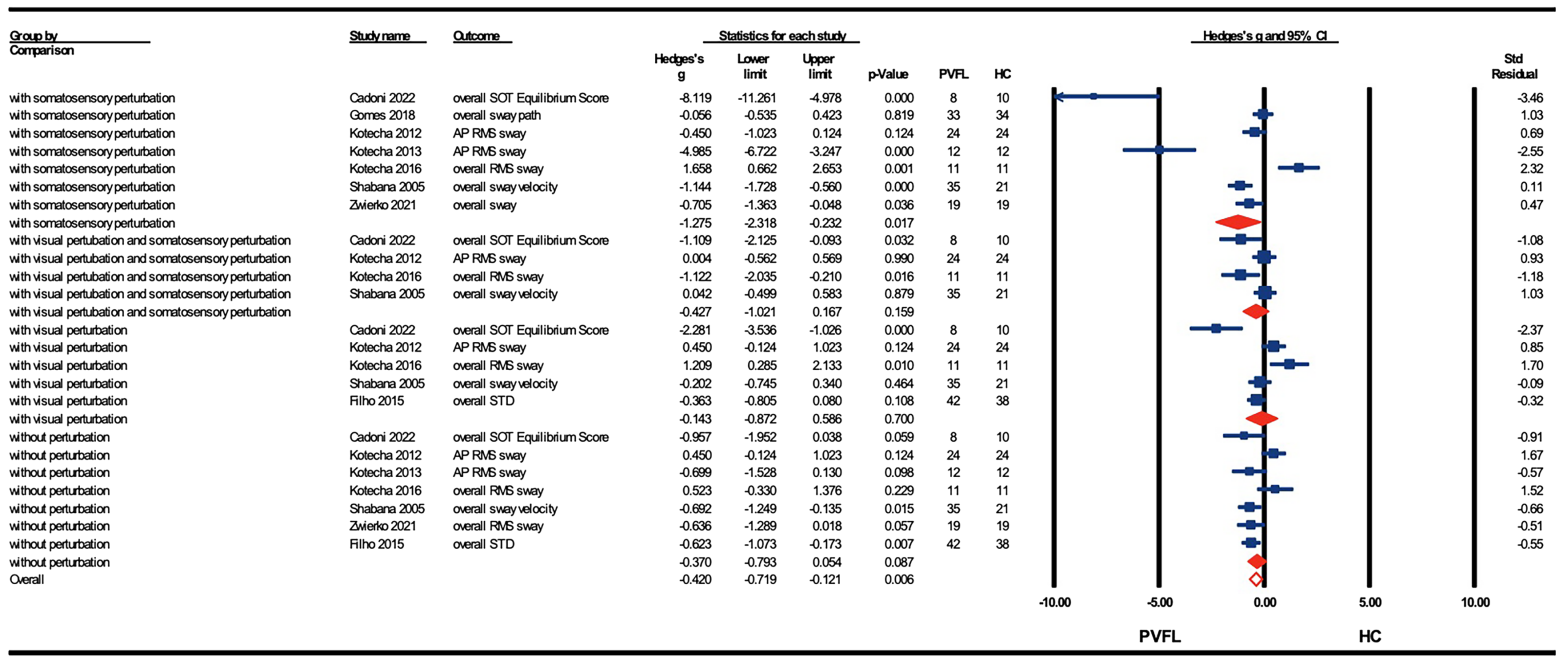
Forest plot displaying the pooled effects of studies examining balance functions under different sensory inputs: (1) with somatosensory perturbation interventions; (2) simultaneously with visual and somatosensory interventions; (3) with visual perturbation interventions; (4) without any perturbation interventions. Each blue line represents an individual study's Hedges’ g and corresponding 95% CI. The size of the square markers reflects the weight assigned to each study in the meta-analysis. The red diamond at the bottom indicates the overall pooled effect estimate, with its width representing the confidence interval. Data suggest variability across studies, with the overall effect indicating a significant reduction in the balance function in PVFL compared to HC. AP, anterior-posterior; RMS, root mean square; STD, standard deviations of the torque moments.

Eight studies were included in the meta-analysis of sensory strategy,^13^^–^^15^^,^^19^^,^^27^^–^^30^ with an overall heterogeneity (*I^2^) index* of 81.4%. Generally, although the overall balance control in people with PVFL without any disturbance was poorer than that of HCs, the difference didn't reach the level of significance according to the result of meta-analysis (Hedges’ *g* = −0.370; 95% confidence interval [CI], [−0.793, 0.054]; *P* = 0.08; Fig. 2).

**Figure 3.**
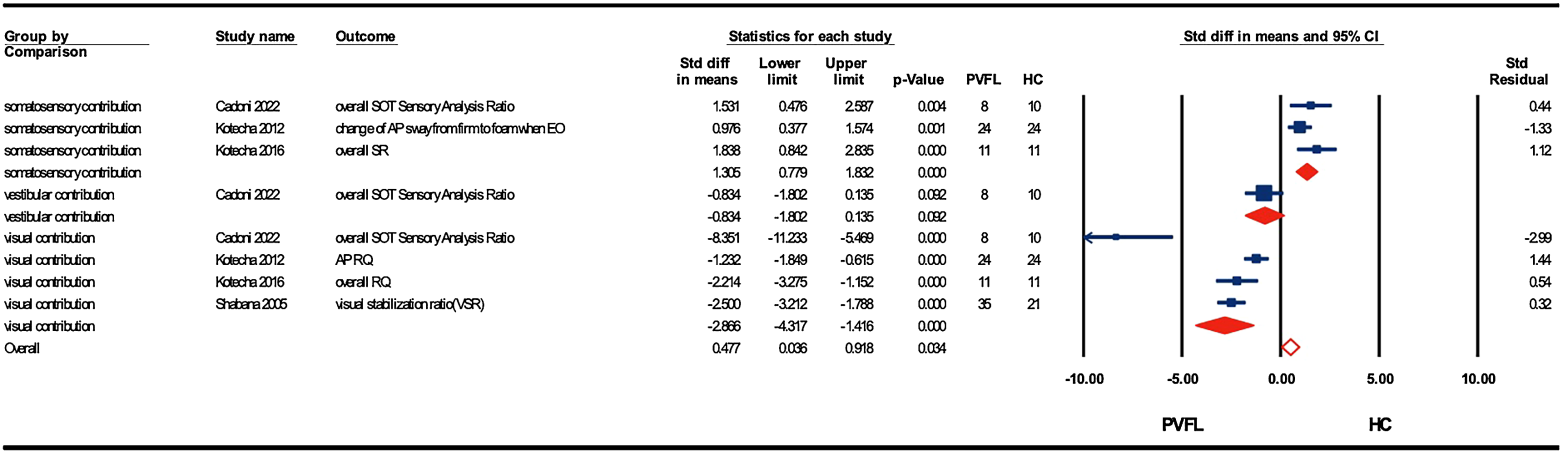
Forest plot displaying the pooled effects of studies examining different sensory contributions to balance function: (1) somatosensory contribution; (2) vestibular contribution; (3) visual contribution. Each *blue line* represents an individual study's Hedges’ *g* and corresponding 95% CI. The size of the square markers reflects the weight assigned to each study in the meta-analysis. The *red diamond* at the bottom indicates the overall pooled effect estimate, with its width representing the confidence interval. Data suggest variability across studies, with the overall effect indicating the overall sensory system's contribution in PVFL subjects is poorer than that of HC. RQ, Romberg quotient; SR, somatosensory ratio.

Seven studies^14^^,^^15^^,^^19^^,^^27^^–^^30^ compared balance under somatosensory system perturbation. When somatosensory input is challenged, the sensory strategy for maintaining balance relies solely on the visual and the vestibular systems, indirectly reflecting the contribution of the somatosensory system. Six studies consistently indicated that PVFL participants had worse balance control than HCs in this situation, as evidenced by increased root mean square center of pressure sway on foam surfaces^15^^,^^27^^–^^29^ and higher oscillation on unstable surfaces.^19^^,^^30^ Only one study reported no significant difference.^14^ The meta-analysis (*I^2^ =* 92.1%) confirmed that PVFL participants exhibited poorer balance than HCs under somatosensory perturbation. (Hedges’ *g* = −1.275; 95% CI [−2.318, −0.232]; *P* = 0.017). Three studies further directly calculated the somatosensory system's contribution to maintaining balance. All studies calculated the somatosensory contribution by measuring the ratio of sway when standing on an unstable surface compared to a firm surface, under open-eye conditions. Kotecha et al.^15^^,^^28^ observed that PVFL patients relied more heavily on somatosensory feedback than HCs, as indicated by increased sway changes from firm to foam surfaces with eyes open and higher somatosensory ratios. Cadoni et al.^19^ mentioned a slightly poorer somatosensory sensory analysis ratio in PVFL compared to HCs. The meta-analysis showed a significantly stronger somatosensory contribution to balance in PVFL compared to HCs (Hedges’ *g* = 1.305; 95% CI [0.779, 1.832]; *P* < 0.001; Fig. 3).

Four studies examined balance function while simultaneously disrupting visual and somatosensory inputs to indirectly assess the contribution of the vestibular system to balance control.^15^^,^^19^^,^^28^^,^^29^ Three studies found no significant difference in balance control between PVFL and HC in this scenario (*P* > 0.05 for all), except for Cadoni's study, which found a significant decrease in the score in the PVFL group (*P* = 0.032). Furthermore, only Cadoni's study directly calculated the vestibular contribution and found it to be similar between groups.^19^ The meta-analysis (*I^2^ =* 60.2%) also further indicated that under these conditions, although the sway data of the PVFL group were lower than those of the HC group, the difference was not statistically significant (Hedges’ *g* = −0.427; 95% CI [−1.02, 0.16]; *P* = 0.159; Fig. 2).

The comparison of the balance function under visual perturbation between PVFL and HC was examined in five studies.^13^^,^^15^^,^^19^^,^^28^^,^^29^ After eliminating visual input, the remaining sensory inputs would rely more heavily on the somatosensory and vestibular systems, which could indirectly reflect the visual system's contribution to balance maintenance. Four studies showed no significant differences between the two groups (*P* > 0.05 for all). Specifically, similar sway velocity^29^ and root mean square^15^^,^^28^ sway were observed for both PVFL and HC groups with eyes closed on a firm surface. Cadoni et al.^19^ also found no significant differences in the equilibrium scores without visual input. Only Filho et al.^13^ reported a significantly higher standard deviation of the trunk movement in PVFL patients compared to HC with rotational and translational dynamic visual stimuli (*P* < 0.05). The meta-analysis (*I^2^* = 80.1%) indicated that, although balance performance was poorer in PVFL, the difference did not reach statistical significance (Hedges’ *g* = −0.143; 95% CI [−0.872, 0.586]; *P* = 0.700; Fig. 2). However, regarding the direct calculation of the visual system's contribution to maintaining balance, four studies consistently reported diminished visual contribution in PVFL compared to HC.^15^^,^^19^^,^^28^^,^^29^ Various metrics were used, including the Romberg quotient,^15^^,^^28^ visual stability ratio,^29^ and sensory analysis ratio.^19^ Despite methodological differences, all metrics compared performance with eyes closed versus eyes open on surfaces lacking somatosensory input. Across four studies (*I^2^* = 88.6%), meta-analysis showed that the visual contribution to balance in PVFL was significantly poorer than in HCs (Hedges’ *g* = −2.866; 95% CI [−4.317, −1.416]; *P* < 0.001; Fig. 3).

#### Motor Strategy

Four studies compared the motor strategy of balance function between PVFL and HC.^14^^,^^16^^,^^19^^,^^32^ Two studies using standing balance tests found that HC had higher passing rates than those with PVFL.^16^^,^^32^ Two studies using the Time Up and Go test reported that PVFL participants had longer Time Up and Go times compared to their age-matched HCs.^14^^,^^16^ One study employed the Motor Control Test to assess the automatic motor reflex responses after unexpected perturbations^19^ and found no significant differences in latency between groups, except for medium forward and backward translations on the left leg.

### Association Between Visual Field Loss and Balance Function

The relationship between visual field and balance function was investigated in nine studies.^15^^,^^29^^,^^30^^,^^33^^,^^35^^–^^39^ Of these, four studies examined the correlation between visual field loss and the motor strategy of balance function,^35^^,^^36^^,^^38^^,^^39^ whereas the remaining seven explored its association with sensory strategy related to balance function.^15^^,^^29^^,^^30^^,^^33^^,^^35^^,^^37^^,^^38^

For motor strategy, Black et al.^39^ observed that a poorer inferior visual field was associated with poorer Time Up and Go performance. However, Finger et al.^36^ found no significant relationship between binocular visual field and Time Up and Go performance, although its time was correlated with visual acuity. De Luna et al.^35^ reported that a worse visual field was linked to increased Root Mean Square sway and jerk during feet-together testing, but not in tandem or semi-tandem conditions. Tokunaga et al.^38^ found no significant association between visual deficits and the Activities-specific Balance Confidence Scale.

The seven remaining studies assessed the relationship between sensory strategy for balance and the visual field.^15^^,^^29^^,^^30^^,^^33^^,^^35^^,^^37^^,^^38^ Under conditions with somatosensory system disturbances, larger monocular^15^ and binocular^29^^,^^37^^,^^38^ visual field loss was a significant predictor of larger somatosensory contribution ratio^15^^,^^38^ and increased postural sway,^30^^,^^33^^,^^35^^,^^37^ although two studies reported no association with balance performance.^15^^,^^38^ Under visual system disturbances, visual field loss was linked to visual contribution ratio^15^^,^^29^^,^^33^^,^^35^^,^^37^^,^^38^ and balance performance.^33^^,^^37^ Specifically, increasing monocular^29^^,^^37^^,^^38^ and binocular visual field loss^15^^,^^33^^,^^35^ exhibited a negative correlation with visual contribution ratio, especially in anterior-posterior direction.^15^ Two studies further explored superior and inferior visual field loss with conflicting findings.^33^^,^^35^ and one found no correlation with balance performance under this situation.^15^ Under vestibular system disturbances, larger monocular visual field deficits were associated with worse balance performance.^37^

## Discussion

This systematic review and meta-analysis comprehensively evaluated the impact of PVFL on balance function, with a particular focus on the independent aspects of sensory and motor systems. Meanwhile, we also explored the associations between the visual field and both sensory and motor strategies. Our findings provide new insights into these relationships. To our knowledge, this is the first review to systematically differentiate the sensory and motor strategies of balance impairment in PVFL, offering a more nuanced understanding that can inform targeted rehabilitation strategies.

### Sensory Strategy of Balance Function

Eight studies assessed the sensory strategy aspect of balance function.^13^^–^^15^^,^^19^^,^^27^^–^^30^ The meta-analysis showed a decline in overall sensory strategy for individuals with PVFL, although the degree of impairment varied across specific sensory systems.

When both visual and somatosensory inputs were perturbed (to isolate vestibular system contribution), three studies^15^^,^^28^^,^^29^ found no significant difference in balance performance between PVFL and HC groups, whereas only one study reported worse balance in the PVFL group under these conditions.^19^ This discrepancy may be attributable to the degree of visual field impairment in Cadoni's study (binocular peripheral visual field ranged from 3.5% to 22.5%). A more detailed analysis of vestibular contribution showed no significant effects, suggesting that the differences were more likely due to other factors—such as motor strategy—rather than vestibular input. Overall, the decline in sensory strategy between the PVFL and HC groups appeared to arise primarily from the integration of the visual and somatosensory systems, rather than from the vestibular system. Therefore we summarized the balance performance of adults with PVFL versus HC under conditions that perturb somatosensory and visual systems separately.

Seven studies^14^^,^^15^^,^^19^^,^^27^^–^^30^ that disrupted somatosensory system input showed poorer balance performance in the PVFL group under this condition, with only one study reporting a similar trend between the PVFL and HC groups.^14^ This might have been attributed to the relatively mild visual field loss in that study compared to others, which may have been insufficient to reveal a measurable deficit in balance function. It is also possible that participants rely more on the motor strategy or on central visual cues,^10^ thereby maintaining balance performance comparable to HCs. The findings primarily confirm that the PVFL groups’ somatosensory systems play an increasingly vital role in maintaining balance.

Five studies manipulated visual input^13^^,^^15^^,^^19^^,^^28^^,^^29^ reported a similar trend between PVFL and HC groups, with only one reporting poorer balance in PVFL individuals during dynamic visual disturbances.^13^ Other studies used static visual perturbation (eyes closed), whereas only one study that employed dynamic perturbation reported poorer balance. Because that PVFL impairs processing of complex, dynamic visual information due to retinal ganglion cell loss,^40^ individuals with PVFL may have had less effective compensatory responses to dynamic visual changes and therefore would perform worse in this situation. Overall, the meta-analysis suggests similar postural control under visual perturbation in PVFL and HC groups. This lack of a group difference likely reflects the predominance of participants with mild visual field loss, which may have limited the ability to detect group-level effects. In addition, the use of somatosensory compensation—greater reliance on proprioceptive input when vision is disturbed to maintain balance—may allow individuals with PVFL to preserve balance performance.

To further evaluate the relative weighting of the visual and somatosensory systems in maintaining balance, direct system-contribution calculations were performed across studies. The second meta-analysis on the system contribution revealed that individuals with PVFL exhibited a poorer visual contribution but a stronger somatosensory contribution compared to HCs. These findings suggest the existence of a compensatory mechanism: specifically, as the visual system is compromised, individuals with PVFL reweight sensory inputs, relying more on somatosensory cues—including the perception of touch, pressure, position, and movement—to maintain balance.

### Motor Strategy of Balance Function

Four studies assessed the motor strategy of balance function. The synthesis revealed that the individuals with PVFL had significantly poorer motor strategy compared to age-matched HCs, suggesting diminished muscle control, coordination, and strength. Previous research^41^ has established a link between physical activity and muscle function. One possible explanation for impaired motor strategy in PVFL is reduced physical activity levels compared to HCs,^42^ which may lead to poorer muscle strength. This aligns with findings that physical activity diminishes as PVFL progresses, reinforcing the observed association between PVFL and impaired motor output in balance function.^43^

### Association Between Visual Field Loss and Balance Function

Nine studies examined the relationship between visual field loss and balance function. The relationship between visual field loss and motor strategy of the balance function remains controversial, with inconsistent findings across four studies. These discrepancies likely reflect the use of different balance measures, each capturing distinct components of motor strategy (e.g., responses to external perturbations, muscle coordination). Studies focusing on different facets of motor control may therefore reach different conclusions. For the two studies investigating VF loss and TUG performance, divergent results may be attributed to differences in the underlying VF impairment. For example, Black et al.^33^^,^^34^^,^^39^ included glaucoma patients with varying degrees of VF loss, whereas Finger et al.^36^ focused on RP patients with severe VF loss. More research is needed to clarify the influence of visual field loss severity, location, and underlying mechanisms on different aspects of motor strategy.

For the sensory strategy, all studies indicated that worse monocular and binocular visual fields were associated with reduced visual contribution and enhanced somatosensory contribution. This suggests that greater PVFL promotes reliance on somatosensory inputs for balance, supporting a compensatory mechanism in PVFL. In contrast, findings on the relationship between visual field loss and overall balance performance were less consistent. The younger age of subjects in the studies by Kotecha and Tokunaga, compared with other cohorts,^30^^,^^33^^,^^35^^,^^37^ may partly explain these divergent discrepancies and suggest that the somatosensory compensation mechanism in PVFL groups could be age-dependent.^15^ Moreover, two studies investigating the impact of superior vs. inferior visual field loss on visual contribution to balance also reached contradictory conclusions, likely due to differences in visual field damage severity. Black's study included a higher proportion of patients without visual field loss than de Luna's study. Clarifying the specific role of the inferior visual field in balance will require larger samples with a wide range and distribution of visual field defects (e.g., lower-left/lower-right defects).

### Clinical Implications

Our findings have important implications for clinical practice. Given the increased reliance on somatosensory input in individuals with PVFL, rehabilitation programs should prioritize somatosensory retraining and compensation strategies. For example, interventions could aim to enhance visual system contribution through neural plasticity^44^ and to strengthen somatosensory input.^45^ To leverage neural plasticity for enhancing visual contributions to balance, several studies have employed non-invasive brain stimulation (NIBS) techniques—such as transcranial direct current stimulation (tDCS) and transcranial alternating current stimulation (tACS)—targeting the visual cortex.^44^ Given the established evidence regarding the cerebellum's critical role in visual-cognitive processing,^46^^,^^47^ future research could also explore the application of NIBS to the cerebellum to evaluate its potential impact on balance function for patients with PVFL.

To enhance somatosensory input, several somatosensory retraining interventions—such as action video game training,^48^ yoga, and Tai Chi^49^—have been developed for visually impaired patients.^50^ However, structured somatosensory retraining specifically tailored for PVFL is still in the early stages of development. To address this gap, the ongoing Glaucoma Rehabilitation With Action Video Games and Exercise (GRADE) trial—a randomized controlled trial comparing action video game training with conventional physical training—is currently investigating the efficacy of these strategies in improving balance and mobility for this population.^48^ Such efforts are essential for the eventual integration into standard care. Furthermore, routine balance assessments in this population should incorporate both sensory and motor evaluations to identify specific deficits. Early identification and targeted intervention may help reduce fall risk and improve the quality of life for individuals with PVFL.

### Limitations and Future Directions

This review has several limitations. First, the substantial heterogeneity observed across studies that may be attributed to variations in the etiology and severity of PVFL, differences in balance assessment protocols, and participant demographics, may cause the unstable meta-analysis results. Although random-effects models were used to mitigate this, these factors may limit the generalizability of our findings. Second, the review focused exclusively on adults with PVFL resulting from eye diseases such as glaucoma and RP, and excluded both children and individuals with PVFL because of neurological conditions such as stroke. Therefore the findings may not be generalizable to pediatric populations or to those with PVFL of neurological origin. Third, our review focused on balance function, limiting discussion on other functional outcomes in PVFL, such as falls.

Despite these limitations, this review has notable strengths. It is the first systematic review to comprehensively summarize balance function in adults with PVFL. Our review achieved a high AMSTAR score (13/16), indicating strong methodological rigor as assessed by the AMSTAR (A MeaSurement Tool to Assess systematic Reviews) checklist, a widely used instrument for evaluating the quality of systematic reviews.^51^ Most studies included in this review focus on the sensory strategy in PVFL, whereas only a few address motor strategy, and conclusions about the relationship between visual field loss and motor strategy remain controversial. Future research should therefore examine the different components of motor strategy in PVFL in more detail. Meanwhile, the association between the location of visual field loss and the visual contribution to balance control also remains unclear. More studies are needed to clarify this relationship and to inform better-targeted visual-rehabilitation approaches. Future reviews should investigate balance function and falls across a wider age range, including both pediatric and geriatric populations, as well as individuals with PVFL resulting from neurological conditions such as stroke or traumatic brain injury.

## Conclusion

In summary, adults with PVFL demonstrate significantly poorer balance function compared to healthy controls, with greater loss of visual field correlating with increased balance impairment. Our findings highlight the critical interplay between visual and somatosensory systems in maintaining postural stability. Clinicians should consider comprehensive balance assessments and interventions that enhance both visual compensation and somatosensory retraining. Further research should focus on exploring the precise relationships between visual field loss and motor/sensory strategy, and developing targeted and evidence-based rehabilitation strategies for this population.

## Supplementary Material

Supplement 1

Supplement 2

Supplement 3
