# Supplementary material for: Balance Performance in People With Peripheral Visual Field Loss: A Systematic Review and Meta-Analysis
Source: Transl Vis Sci Technol. 2026 Jul 8;15(7):10. doi: 10.1167/tvst.15.7.10 (PMC13355389; doi:10.1167/tvst.15.7.10)
Supplement: Supplement 2 [file tvst-15-7-10_s002.pdf]

| | | | |
|---|---|---|---|
| **Databases** | PubMed (Medline), AMED, Scopus, CINAHL, Web of Science | | |
| **Restrictions** | English and Chinese, 2003-2023 | | |
| **Themes** | #1 Peripheral visual field loss | #2 Balance performance | #3 |
| **MeSH Terms** | Peripheral visual field loss, vision loss, peripheral vision loss, visual field damage, visual field defect, Glaucoma, Retinitis pigmentosa, RP | Balance, Postural sway, Postural control, Postural stability, Stabilization, Equilibrium, Mobility, Fall | #1 AND #2 |
