# Supplementary material for: Balance Performance in People With Peripheral Visual Field Loss: A Systematic Review and Meta-Analysis
Source: Transl Vis Sci Technol. 2026 Jul 8;15(7):10. doi: 10.1167/tvst.15.7.10 (PMC13355389; doi:10.1167/tvst.15.7.10)
Supplement: Supplement 3 [file tvst-15-7-10_s003.pdf]

Methods	Balance Tests
Sensory strategy	1. Sensory Organization Test<br>2. Modified Clinical Test of Sensory Interaction in Balance<br>3. Clinical Test of Sensory Interaction in Balance<br>4. Postural sway test on firm/foam surfaces with eyes open/eyes closed/virtual reality equipment
Motor strategy	1. Time up and Go Test<br>2. Motor Control Test<br>3. Three 30-second timed stands<br>4. One-legged balance test
